# Thoracic Wounds—Infected Haemothorax

**Published:** 1918-11

**Authors:** 


**Thoracic Wounds—Infected Haemothorax.** Wm. Hutchinson, Brit. M. J., Feb. 16, reports a total of 450 military cases with only 29 deaths. In uninfected haemo- and pneumothorax, aspiration should be done and is usually sufficient if the dullness does not exceed two fingers' breadths. If the effusion of blood is massive, the pleura should be opened as soon as possible and the liquid blood and clots removed and the cavity irrigated and closed. Open haemothorax should be closed as soon as possible. Early diagnosis of infection should be made, pleurotomy with resection of rib practiced, blood and clots removed, and irrigation should be done with an antiseptic leaving a small quantity in place and closing the cavity. In case of development of infection after aspiration or recrudescence after pleurotomy, the pleura should be opened promptly. Attempt at extraction of a foreign body should not be made immediately and, in the case of British troops, not till the patient has reached England.

